# T‐PRP‐DAT Gel: A Novel Material Promotes Adipose Tissue Regeneration

**DOI:** 10.1111/jocd.70045

**Published:** 2025-02-18

**Authors:** Mengmeng Hou, Jiezhang Tang, Yajie Guo, Han Peng, Baoyan Liang, Yi Cheng, Zhaoxiang Zhang, Siming Wei, Chenggang Yi, Huichen Li

**Affiliations:** ^1^ Department of Plastic and Reconstructive Surgery Xijing Hospital, Fourth Military Medical University Xi'an China; ^2^ The Second Affiliated Hospital of Zhejiang University College of Medicine Hangzhou China

**Keywords:** adipose tissue regeneration, angiogenesis, decellularized adipose tissue, interpenetrating polymer network, platelet‐rich plasma

## Abstract

**Background:**

Decellularized adipose tissue (DAT) has emerged as a promising tissue‐specific regenerative platform for soft tissue augmentation and reconstruction. Hydrogels are a widely used DAT scaffold format for their injectability and porous structure. While unstable structure and poor vascularization limit the adipose tissue regeneration of DAT gels, this yields significant clinical necessity for solutions to this problem.

**Methods:**

Based on collagen‐fibrin interpenetrating, we developed an injectable thermosensitive DAT/PRP interpenetrating polymer network (t‐DP gel/t‐DPI) with the composition of DAT and temperature‐controlled platelet‐rich plasma (t‐PRP). The same volume of t‐DP gel and DAT gel were transplanted in a mouse model, and graft volume, weight, adipose tissue regeneration rate, and vascularization were compared.

**Results:**

The t‐DPI showed reinforced stability for the interpenetrating polymer network (IPN) of collagen‐fibrin and sustained release of growth factors from the t‐PRP, resulting in improved graft volume, weight, adipose tissue regeneration, and vascularization.

**Conclusions:**

Compared with traditional DAT gel, t‐DP gel promotes adipose tissue regeneration by promoting angiogenesis and stability, and t‐DP gel has great potential for future applications in the field of plastic surgery for its simple preparation and regeneration ability.

## 1 Introduction

Autologous fat grafting is now a commonly used technique for soft tissue reconstruction [1, 2]. Howere, graft retention, which can range from 25% to 80% relative to the original volume, remains less than ideal [3, 4]. Furthermore, some patients do not have enough autologous fat for transplant, and nonregenerative therapies such as biodegradable polymer‐based fillers fail to offer the regenerative framework required for functional improvement, limiting sustained efficacy. Decellular biological scaffolds have good tolerance to the host, effectively reducing immune rejection reactions and promoting structural remodeling [5]. The decellularized scaffolds of natural tissues are freeze‐dried, ground, and digested with enzymes. This process eliminates antigenic cellular components while preserving significant compositional, structural, and biomechanical cues of the native tissue microenvironment [6], The decellular matrix hydrogels retain the structural and stimulating characteristics of the hydrogel's responsiveness. Decellularized adipose tissue (DAT), due to its ability for adipose tissue regeneration and allogeneic application [7, 8], is becoming a promising substitute for autologous fat for soft tissue reconstruction.

DAT, which is made up of collagens, glycosaminoglycans, laminin, and elastin, can be generated from human adipose tissue waste [9, 10]. Then, DAT can be employed as a biological scaffold in many forms to match the contributory effects in tissue regeneration and DAT hydrogel is one of the most commonly used for its injectability and porous structure [11]. The easy preparation process for DAT hydrogel results in a fluid material at 4°C, which gels at 37°C. DAT scaffolds offer a favorable milieu for soft tissue regeneration, promoting blood vessel formation and adipocyte proliferation, according to in vivo research conducted using mouse models [12]. Notably, a recent clinical study reported that an injectable DAT formulation showed no major adverse events and supported volume retention and adipogenesis at 4 months post delivery subcutaneously within the dorsal wrist of healthy human volunteers [13]. These effects may be mediated by the presence of growth factors or other bioactive components within the decellularized ECM. However, neovascularization of DAT hydrogel is still unsatisfactory, and the unstable structure of DAT hydrogel would lead to rapid collapse after transplantation, which is unfavorable to cell infiltration and regeneration. So, it yields a significant clinical necessity for solutions to this problem.

Platelet‐rich plasma (PRP), which comprises platelets, several growth factors, and a fibrin network, exhibits a beneficial effect on tissue regeneration and wound healing due to its strong promoting effect on angiogenesis [9]. Here, we developed an injectable thermosensitive DAT/PRP interpenetrating polymer network (t‐DP gel/t‐DPI) with the potential to address the limitations mentioned above the unstable structure of DAT hydrogel. The t‐DP gel is composited of DAT and temperature‐controlled platelet‐rich plasma (t‐PRP), using a novel convenient PRP preparation method without thrombin and anticoagulant, and the activation of PRP and the formation of the fibrin network were controlled by temperature change. Both DAT and PRP alone can form hydrogels when the temperature rises to 37°C. Significantly, collagen in DAT and fibrin in t‐PRP may also form a stable interpenetrating network hydrogel based on collagen‐fibrin interpenetrating, suggesting a more stable structure in the t‐DP gel. Moreover, in the t‐DP gel, multiple growth factors from t‐PRP could be sustainably released. Compared with DAT gel, the t‐DP gel may have reinforced stability and sustained release of growth factors, leading to an increase in adipose tissue regeneration ability.

To prove this hypothesis, the stability of DAT gel and t‐DP gel was evaluated by their shape‐keeping ability and liquid precipitation over time. Utilizing scanning electron microscopy (SEM) examination, the collagen‐fibrin interpenetrating polymer network (IPN) in t‐DP gel was verified. ELISA kits were used to identify the continuous release of growth factors from t‐PRP in t‐DP gel. To verify the in vivo regeneration of hydrogel, the same volume of t‐DP gel and DAT gel was transplanted in a rat model. Then, the graft volume, weight, and adipose tissue regeneration rate were evaluated and compared. The graft volume, weight, and adipose tissue regeneration rate of the t‐DP gel were greatly improved, as was the DAT gel, which showed a poor adipose tissue regeneration rate as previously reported. Angiogenesis evaluated by immunofluorescence staining of CD31 and mature blood vessels evaluated by immunofluorescence staining of α‐SMA in t‐DP gel increased significantly compared with DAT. Compared with traditional DAT, the t‐DP gel promotes adipose tissue regeneration through reinforced stability and sustained release of growth factors that promoted angiogenesis.

The t‐DP gel has great potential applications in many fields for its simple preparation, injectable bioactivity, and regeneration ability. The t‐DP gel could be transformative is in various wound healing for its ability to promote angiogenesis and tissue regeneration. Its thermosensitive properties allow for easy injection and controlled release of growth factors, which could accelerate wound closure and reduce scarring. This is particularly beneficial for patients with chronic wounds or those who have undergone extensive surgeries where traditional wound management techniques may be less effective [9]. In the field of plastic and reconstructive surgery, t‐DP gel can be used for soft tissue augmentation and contouring. Its stable structure and regenerative capabilities make it an ideal material for breast reconstruction, facial rejuvenation, and other cosmetic procedures. The gel's ability to enhance tissue regeneration and vascularization can lead to more natural and long‐lasting results, reducing the need for additional surgeries and improving patient satisfaction [14]. Moreover, the gel's immunomodulatory properties, such as promoting M2 macrophage polarization, can help in reducing inflammation and enhancing the body's natural healing response. This makes it a promising candidate for use in postsurgical recovery, where minimizing inflammation and promoting tissue repair are critical for optimal outcomes [15]. Overall, multifunctional nature of the t‐DP gel positions it as a valuable tool in regenerative medicine, offering innovative solutions to challenges in wound healing, soft tissue reconstruction, and postsurgical recovery. Its potential applications highlight the significance of this research in advancing clinical practices and improving patient outcomes.

## 2 Materials and Methods

### 2.1 Preparation of DAT Gel

According to the previously suggested method [9, 16], human fat extract was decellularized, freeze‐dried, and ground to obtain DAT powder. DAT powder is digested in HCl and pepsin solutions. Stir the mixed solution at room temperature until DAT is fully dissolved. Then, adjust PH to 7.4 with NaOH and PBS to obtain a DAT pre‐gel solution. After the pregel solution is incubated at 37°C, the pregel solution turns into water.

### 2.2 Preparation of t‐PRP

The temperature‐controlled PRP was prepared using the methods reported in previous research reports [17], and some adjustments were made. Collect venous blood from healthy adult volunteers with a precooled vacuum blood collection tube that only contains blood separation gel and does not contain anticoagulant. Centrifuge whole blood for 10 min (200 g, 4°C), then transfer the plasma to a new precooled blank tube and centrifuge again for 10 min (1550 g, 4°C). Discard the supernatant, the bottom plasma and precipitated platelets are inactive t‐PRP and are temporarily stored at a low temperature for further use. One milliliters of inactive t‐PRP can be obtained from 10 mL of whole blood. Activated t‐PRP can be obtained by incubating at 37°C for 15 min.

### 2.3 Preparation of Thermosensitive PRP/DAT Interpenetrating Polymer Network Hydrogel (t‐DP Gel)

Mix the inactive t‐PRP and DAT pre‐gel with the spiral syringe and Luer Lock connector at low temperature to obtain t‐DP pre‐gel. The t‐DP gel hydrogel containing 50% t‐PRP (v/v) was prepared for further study. By adjusting the concentration of DAT pre‐gel before mixing, the final concentration of DAT in the above hydrogel is controlled at 8 mg/mL.

### 2.4 Characterization of the Interpenetrating Polymer Network in t‐DP Gel

Using scanning electron microscopy (SEM) analysis to identify the microstructure of hydrogel and validate the formation of the interpenetrating network structure of t‐DP gel. Place the hydrogel (a fresh sample without freeze drying or gold plating) on the temperature control sample rack and cool it from room temperature to −20°C. The morphology was observed and captured using a Phenom World, Phenom Pro, The Netherlands desktop microscope at 15 kV.

### 2.5 In Vitro Growth Factor Release of t‐DP Gel

To determine the GFs release behavior of hydrogels, 1 mL of DAT and 1 mL of t‐DP gel were incubated with 1 mL of PBS at 37°C. Collect PBS (250 μL) at 1, 2, 4, 6, 12, 24, 48, 72, and 96 h, at the same time, replace with fresh PBS. The secretion of various growth factors was measured using VEGF ELISA kit (Beyotime, PV963, China), and PDGF‐BB ELISA kit. Both DAT and t‐DP gel groups consists of 10 samples, with VEGF and PDGF‐BB measured for each group, and five samples per indicator. At each time point (1, 2, 4, 6, 12, 24, 48, 72, and 96 h), measurements of VEGF and PDGF‐BB are repeated from five samples in each group, and each experiment is repeated 3 times.

### 2.6 Evaluation of t‐DP Gel and DAT Gel in a Mouse Model

The 500 μL t‐DP gel and DAT gel, which were stored at 4°C, were injected bilaterally into the subcutaneous dorsal side of 8‐week‐old female mice using a No. 16 blunt head cannula with a single‐point injection technique (1graft for 1 mouse). After injection, the mice were heated with an electric blanket and reheated to about 37°C. During this process, t‐DP gel and DAT gel transformed the fluid into hydrogel. Forty mice were selected as recipients and were randomly divided into two groups for grafts of t‐DP gel or DAT gel, with 20 mice in each group. The mice were maintained at 25°C in a standard breeding environment. Mice of t‐DP gel and DAT group were anesthetized by intraperitoneal injection of sodium pentobarbital (50 mg/kg) at 4 and 12 weeks post transplantation (10 mice for each time point). Then, 10 transplanted t‐DP gel and DAT tissue samples were collected after transplantation at 4 and 12 weeks, and the volume and weight of all grafts were evaluated and recorded. Afterwards, all grafts were fixed in 4% paraformaldehyde, embedded in paraffin blocks, and cut into paraffin sections. Some were treated and stained with hematoxylin and eosin (H&E). Immunofluorescence staining, applying anti‐CD31 and α‐SMA antibodies evaluate angiogenesis.

### 2.7 Statistical Analyses

Statistical analysis was performed using the Prism software (GraphPad Software, GraphPad Prism version 6.02, USA). Data from triplicate experiments were expressed as means ± standard deviation (M ± SD). The *t*‐test, or one‐way analysis of variance, was used to determine the statistical significance of differences, and the statistically significance was determined at *p* < 0.05.

## 3 Results

### 3.1 The t‐DP Gel Is Injectable and Thermosensitive, and the Formation of an Interpenetrating Collagen‐Fibrin Polymer Network Reinforced the Stability of the t‐DP Gel

**SCHEME 1:**
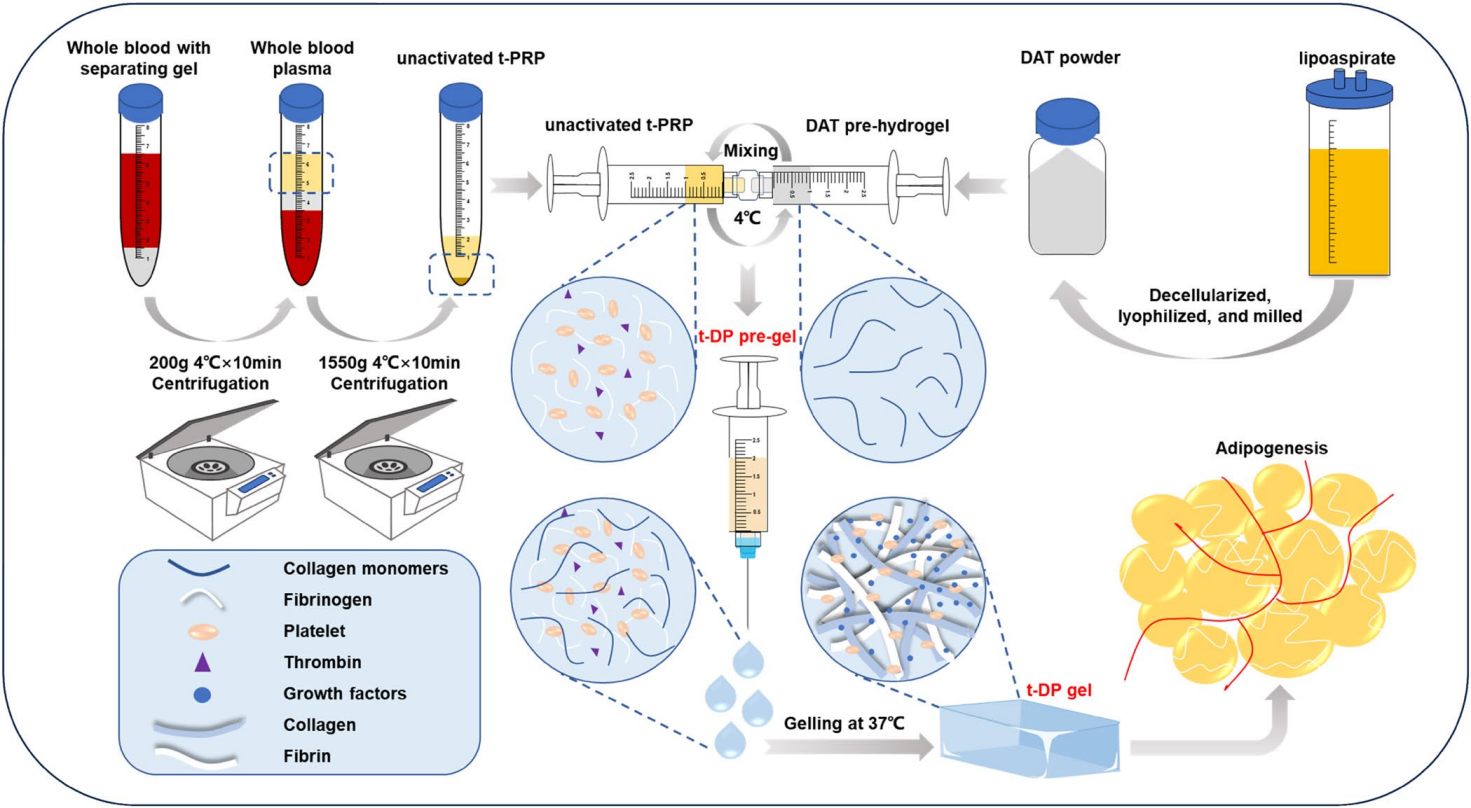
Preparation, formation process and application of thermosensitive DAT/PRP interpenetrating polymer network (t‐DPI) hydrogel. This figure illustrates the process of preparing Platelet‐Rich Plasma (PRP) from whole blood. This gel is rich in platelets and growth factors and is capable of promoting tissue repair.

**FIGURE 1:**
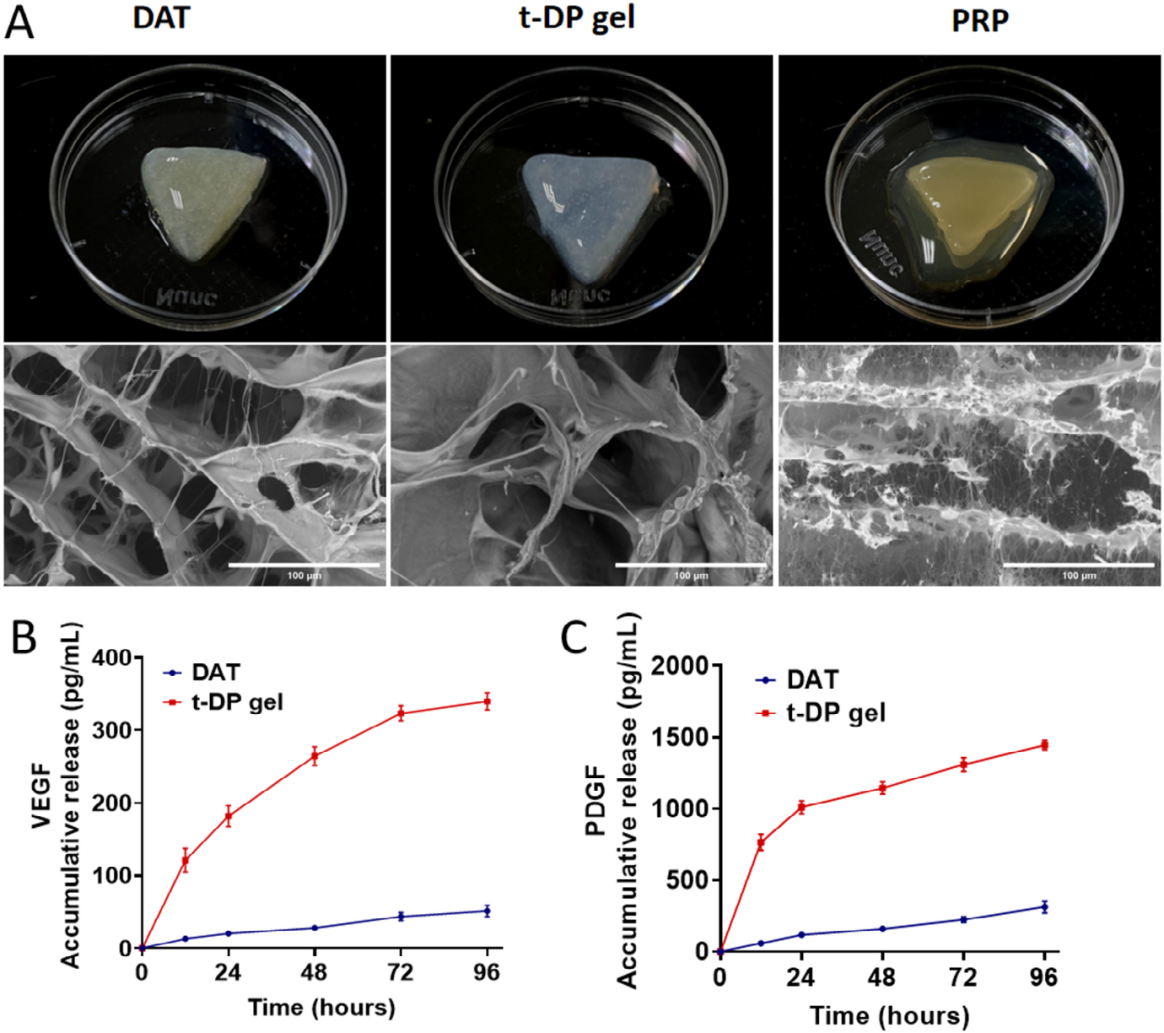
Characterization of hydrogel. (A) Photographs of t‐PRP, DAT and t‐DP hydrogels at 37°C. Typical microstructure images of t‐PRP, DAT, and t‐DP hydrogels detected by SEM. (B) Growth factors release of t‐PRP and t‐DP hydrogels detected by ELISA. Release curve of VEGF in vitro. Compared with t‐PRP hydrogel, t‐DP hydrogel showed sustained release of VEGF (*p* < 0.05). (C) Growth factors release of t‐PRP and t‐DP hydrogels detected by ELISA. Release curve of PDGF in vitro. Compared with t‐PRP hydrogel, t‐DP hydrogel showed sustained release of PDGF (*p* < 0.05).

For the preparation of t‐DP gel, the nonactivated t‐PRP and DAT pregel are mixed at low temperatures to obtain t‐DP gel, which can be gelled and activated at 37°C (Scheme 1). As shown in Figure 1A, after DAT gel, t‐DP gel, and t‐PRP are injected into the triangular mold and placed at 37°C for 5 min, the shape of t‐PRP cannot be maintained, and the liquid falls off, while DAT gel and t‐DP gel remain triangular. In addition, compared with DAT gel, t‐DP gel can better maintain the three‐dimensional triangular shape. Although DAT hydrogel also retains a certain shape, it has collapsed, which is mainly manifested in the obvious enlargement of its triangle and the obvious thinning of its thickness, which is due to poor stability and the occurrence of tiling. As an injectable hydrogel, t‐DP gel has good thermal sensitivity and formability and is very suitable for soft tissue reconstruction.

Apply fresh samples that have not been freeze‐dried or gilded to SEM analysis to obtain more realistic structural images (Figure 1A). The fibrin polymer network in t‐PRP is shown in higher magnification images. DAT hydrogel shows an irregular structure and interconnection pores of different sizes. Although collagen I is the main component of DAT hydrogel, there are many peptides and small molecular weight peptide fragments. The microstructure of DAT hydrogel and collagen I is still different. 3D morphology with smaller and more uniform pores was observed in t‐DPI hydrogel. In the dense network of t‐DPI hydrogel, a fibrin network was found on the pore junction and wall, indicating the formation of an interpenetrating polymer network (IPN).

### 3.2 The t‐DP Gel Showed Sustained Release of VEGF and PDGF, Indicating Its Promoting Effect on Vascularization

Angiogenesis is crucial for tissue regeneration [18, 19], and multiple studies have shown that PRP has a good ability to promote angiogenesis [20, 21]. Our previous research also found that interpenetrating network hydrogels can achieve sustained release of growth factors [9]. In this study, we detected the main growth factors (GFs) in PRP, including vascular endothelial growth factor (VEGF) and platelet‐derived growth factor (PDGF), which are related to angiogenesis ability. VEGF is considered a key dynamic molecule in angiogenesis, and VEGF interacts with VEGFR2 expressed on the surface of resting endothelial cells, enhancing VEGF binding activity and signal transduction through VEGFR2 [22]. Under VEGF stimulation, endothelial cells differentiate and promote angiogenesis. PDGF participates in angiogenesis, immune regulation and tissue regeneration remodeling. PDGF‐B recruits pericytes around newly formed blood vessels, which further stabilize them by secreting angiopoietin‐1 (Ang‐1) [23]. Further determination of the VEGF release curve and detection of PDGF using the ELISA method. As shown in Figure 1B,C, compared with DAT gel, t‐DP gel shows sustained release of growth factor, and its growth factor level is always higher. On the one hand, the above results show that the formation of an interpenetrating polymer network in t‐DP hydrogel may enable the growth factor to achieve sustained release. On the other hand, t‐DP hydrogel may have better angiogenesis regeneration potential than traditional DAT gel due to sustained release of growth factors.

### 3.3 Compared to DAT Gel, the Graft Volume, Weight of t‐DP Gel Were Greatly Improved

**FIGURE 2:**
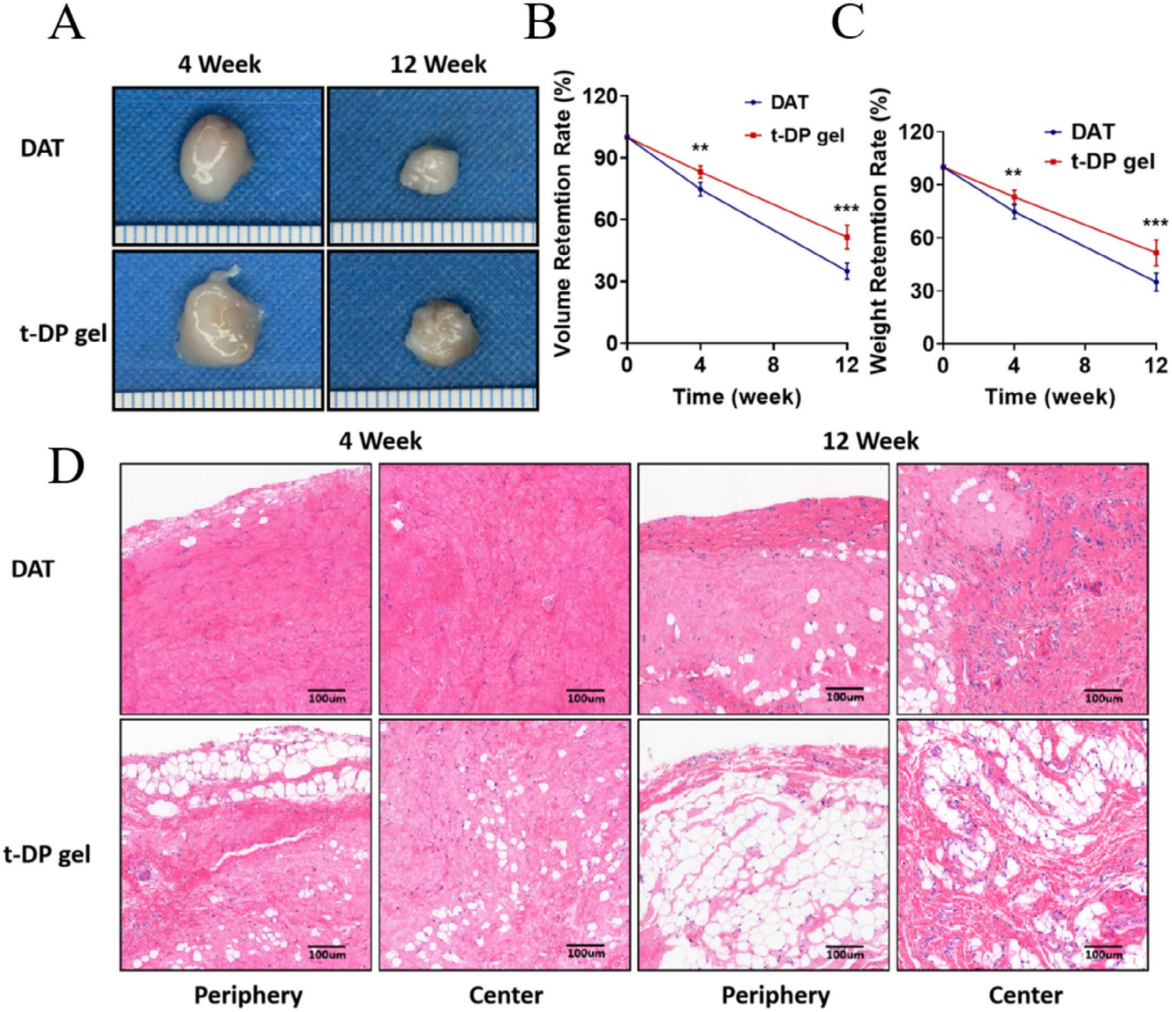
Retention rates of t‐DP and DAT hydrogels and Histologic examination of t‐DP and DAT hydrogels at 4 and 12 weeks. (A) Representative images of t‐DP and DAT hydrogels graft at 4 and 12 weeks. (B) The volume retention rate of the t‐DP hydrogel are higher than DAT hydrogel (*p* < 0.01). (C) The weight retention rate of the t‐DP hydrogel are higher than DAT hydrogel (*p* < 0.01). (D) Tissues were stained with hematoxylin and eosin. Representative photographs of the grafted t‐DP gel and DAT hydrogel at 4 and 12 weeks. DAT gel and t‐DP gel had a nascent region of fat‐like structure, and the regeneration region of t‐DP gel was much higher than that of the DAT group. The new areas of fat‐like structures were surrounded by more homogeneous structures. ** means *p* < 0.01; *** means *p* < 0.01.

The volume and weight retention rate of the graft are very important, and the evaluation of the percentage of graft area that has been reshaped into adipose tissue indicates the regeneration and remodeling of adipose tissue. Grafts of DAT gel and t‐DP gel were harvested at Weeks 4 and 12, and volume and weight retention rates and adipose tissue regeneration were evaluated (Figure 2A). Compared with DAT gel, the graft volume and weight of t‐DP gel increased by about 30% and 35% at Weeks 4 and 12, respectively (Figure 2B,C). This is in line with our expectations because the previous results show that the t‐DP gel has better stability and can better maintain the shape of the gel without rapid collapse. From a microscopic perspective, stable hydrogels can better maintain the porous microstructure and are more conducive to the infiltration of macrophages, mesenchymal stem cells, thus creating better opportunities for adipose tissue regeneration, and obtaining better retention volume and retention rate. As shown in Figure 2D, HE staining showed that at Week 4, both the marginal and central portions of the DAT hydrogel exhibited a more homogeneous and dense structure, with the presence of a small amount of cellular infiltration and no obvious inflammatory reaction present. Adipose‐like structures can be seen in a few marginal areas, and the small size of the adipose‐like cells suggests possible neoplastic adipose tissue. In contrast to the DAT gel, the t‐DP gel group also showed a more homogeneous structure in the marginal and central regions and no obvious inflammatory reaction existed, but the fibers were not densely arranged. Notably, more fat‐like structures were present in the t‐DP gel group and were not only confined to the margins, but were also widely present in the central region. We observed that the adipose‐like cells at the margins were larger in size and existed in a continuous distribution over a larger area, whereas the adipose‐like cells in the central region were smaller in size and showed a scattered distribution, suggesting that perhaps adipose regeneration in the marginal region existed earlier and had already progressed toward greater maturity and was close to normal adipose tissue, whereas the adipose regeneration process in the central region started later and was undergoing the adipose neovascularization The center is undergoing adipose regeneration, which started later. At Week 12, both the edge and center regions of the DAT gel exhibited altered homogeneity, with some regions becoming less dense and accompanied by the apparent neogenesis of fat‐like structures. Compared to Week 4, the number of cellular infiltrates, especially in the central region, was significantly higher, and the neoplastic region of adipose‐like structures was significantly increased, showing a scattered distribution, but the overall percentage was still low. The outermost rim area of the t‐DP gel group was essentially completely filled with adipose‐like structures, which were close to the normal adipose tissue structure, and the number of cellular infiltrations in the rim was lower due to the possible completion of adipose regeneration. The central area was no longer a scattered distribution of adipose‐like structures, but showed a continuous distribution over a larger area, exhibiting the structural features of mature adipose tissue and a higher number of cellular infiltrations, suggesting that a cell‐mediated adipose regeneration process may be underway. In summary, the results showed that both DAT gel and t‐DP gel had a nascent region of fat‐like structure, and the regeneration region of t‐DP gel was much higher than that of the DAT group. The new areas of fat‐like structures were surrounded by more homogeneous structures, which were probably collagen fibers of DAT origin, and there was a gradual change from homogeneous to dense.

### 3.4 Compared to DAT Gel, Thinner Graft Margin Envelope and Significantly Higher Adipose Tissue Regeneration With t‐DP Gel

**FIGURE 3:**
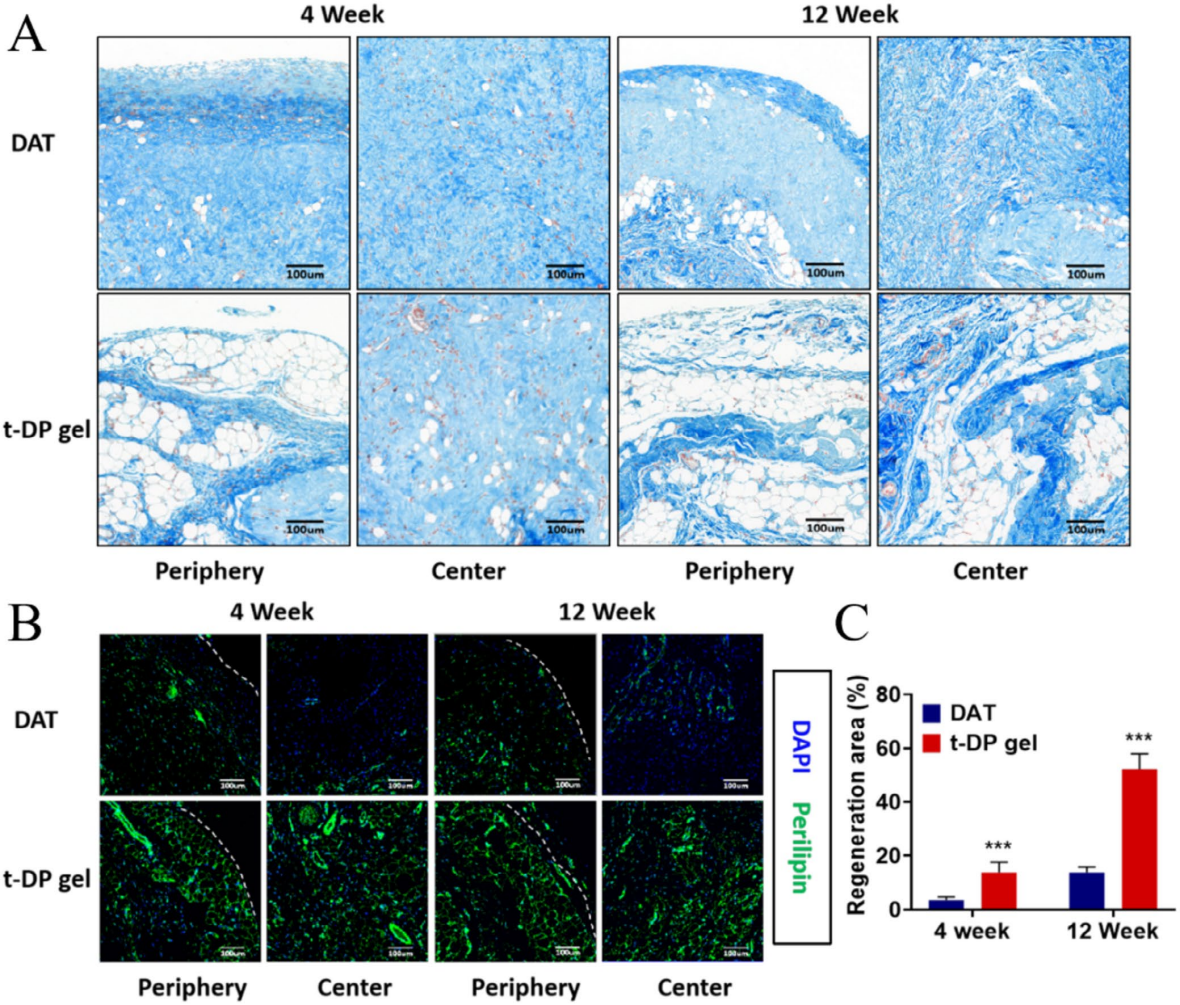
Degree of encapsulation and adipose tissue regeneration rate of t‐DP and DAT hydrogels at 4 and 12 weeks. (A) The envelope of adipose tissue graft margins was thicker in the DAT gel group, and thinner in the t‐DP gel group at Weeks 4 and 12. (B) Tissues were stained with DAPI and perilipin, and adipose tissue regeneration was higher in the t‐DP hydrogel group than in the DAT hydrogel group at both Weeks 4 and 12. (C) Regeneration area statistics. *** *p* < 0.001.

As shown in Figure 3A, Masson staining confirmed that the collagenous tissue surrounding the neoplastic areas of the adipose‐like structures was indeed collagenous, which was consistent with the performance of HE. Compared with the DAT gel, the t‐DP gel group had more fat‐like structures at both Weeks 4 and 12. Notably, the presence of a thicker periplasmic structure in the marginal portion of the DAT group was clearly detected by Masson staining, and the periplasmic structure was not yet dense at Week 4, with an increase in structural densification at Week 12. In contrast, only a thin envelope structure was present in the t‐DP gel group. Since the envelope is an important product of foreign body reaction, the above results are highly suggestive that the addition of t‐PRP can significantly reduce the foreign body reaction brought about by DAT and contribute to fat regeneration, but the specific mechanism is not clear and deserves further in‐depth investigation. In addition, the wrapping of the envelope was unfavorable to cellular infiltration and nutrient exchange, which may be an important reason for the enhanced fat regeneration in the t‐DAT‐PRP (t‐DP gel) hydrogel group at the margins compared with the DAT group.

Since both HE and Masson's histological evaluations evaluated adipose regeneration from the morphological aspect, to further evaluate adipose neogenesis, we performed immunofluorescence staining for the lipid droplet‐encapsulated protein Perilipin, which is an important index for evaluating adipose neogenesis. It was found that, consistent with the histological evaluation by HE and Masson, the Perilipin‐positive adipose tissue regeneration of the DAT gel at Week 4 was very low, with fat regeneration present in only a few areas, with a rate of regeneration of < 5%, and was present only at the marginal locations of the grafts, with weak Perilipin‐positive signals in the central areas(Figure 3B,C). At Week 12, the percentage of Perilipin‐positive adipose regeneration in the DAT gel increased significantly, but was still < 20%. The area of Perilipin‐positive adipose tissue regeneration also expanded from the margins to the interior of the grafts, beginning to induce adipose regeneration in the deeper layers of the grafts. It indicates that fat regeneration of the hydrogel occurred in different locations and at different levels. Consistent with expectations, the percentage of Perilipin‐positive adipose regeneration in t‐DP gel was significantly increased at both Week 4 and Week 12 compared to DAT gel. Notably, the outermost marginal region of the t‐DP gel group was essentially completely filled with Perilipin‐positive adipocytes, which were close to the normal adipose tissue structure. The central region is no longer a scattered distribution of Perilipin‐positive adipocytes but shows a continuous distribution over a large area, exhibiting the structural characteristics of mature adipose tissue.

Due to the fact that early fat regeneration mainly relies on the infiltration of local cells, while late fat regeneration is likely mediated by the infiltration of blood system‐derived cells mediated by neovascularization [24]. The early performance of fat regeneration is consistent with our previous research and hypotheses. Because the t‐DP gel in the early stage can maintain a better porous structure, local cells may be able to better and more infiltrate into the deeper area of the hydrogel, so the ability of early fat regeneration is stronger. In the late stage, infiltrating cells and slow‐release growth factors may better promote angiogenesis, which will be beneficial for blood cell‐mediated fat regeneration.

### 3.5 The Sustained Release of Growth Factors in t‐DP Gel Leads to an Increase in Vascularization

Angiogenesis is a key factor in adipose tissue regeneration and DAT remodeling [25]. Previous experiments have shown that t‐DP gel can continuously release growth factors. We also found that, compared with DAT gel, t‐DP gel significantly improved the graft volume, weight, and adipose tissue regeneration rate. Therefore, we speculate that compared with DAT gel, the vascularization of t‐DP gel may increase, which is an important reason for the enhancement of adipose tissue regeneration and remodeling.

**FIGURE 4:**
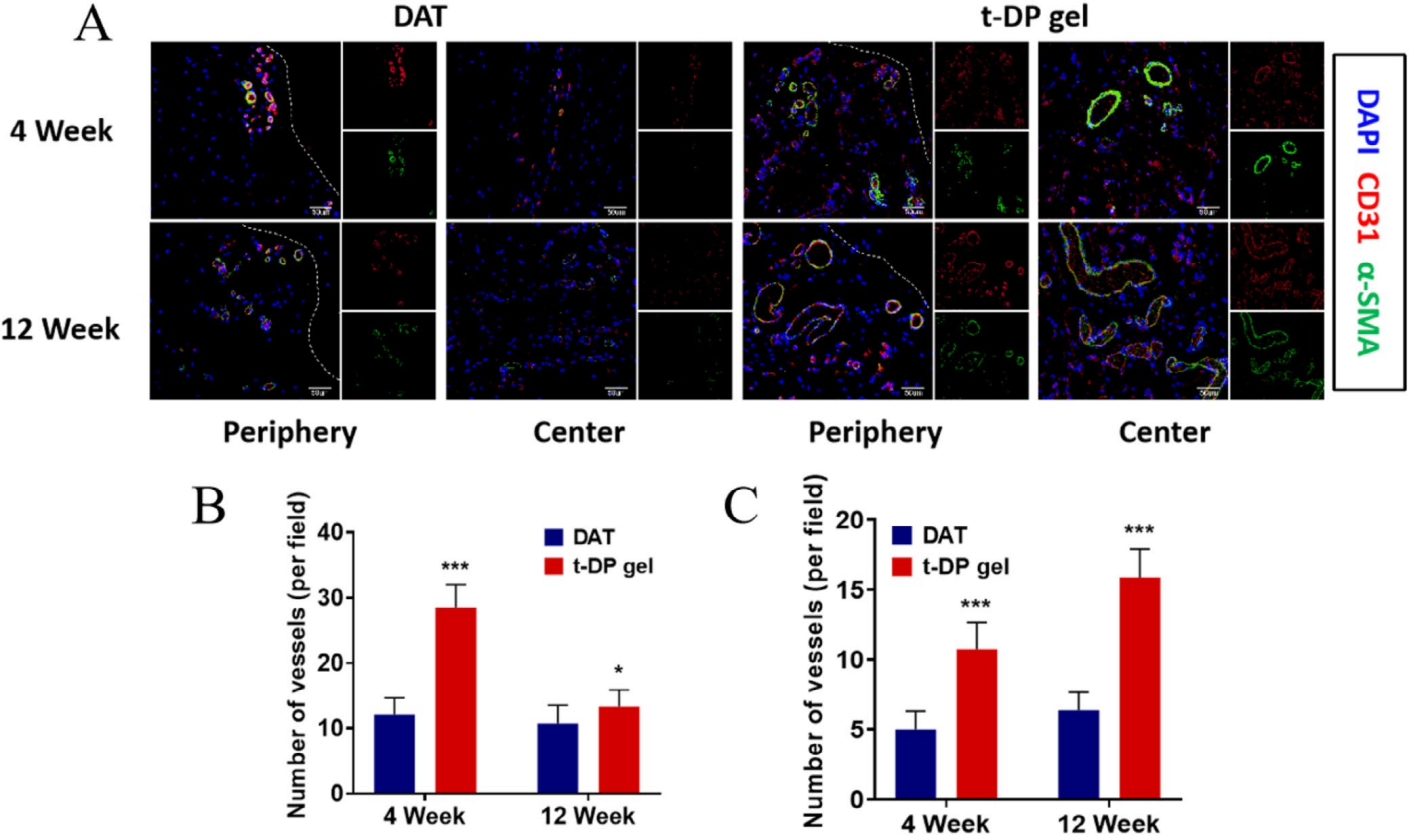
Evaluation of neovascularized capillary density in grafted t‐DP and DAT hydrogels. (A) Representative images of immunofluorescence CD31 and α‐SMA of t‐DP and DAT hydrogels graft at 4 and 12 weeks. (B) Changes in the number of CD31‐positive vessels in the t‐DP hydrogel and DAT hydrogel groups at weeks 4 and 12. (C) Changes in the number of α‐SMA‐positive vessels in the t‐DP hydrogel and DAT hydrogel groups at weeks 4 and 12. * *p* < 0.05; *** *p* < 0.001.

Immunofluorescence staining of CD31 was performed to evaluate the total number of blood vessels, and immunofluorescence staining of α‐SMA and CD31 jointly marked the number of mature blood vessels (Figure 4A). We found that in the DAT gel group, the number of CD31 positive blood vessels did not change significantly from the 4th week to the 12th week, but in the t‐DP gel group, the number of CD31 positive blood vessels decreased to some extent from the 4th week to the 12th week, but the number of blood vessels was higher than that in the DAT gel group, especially at the 4th week, the total number of blood vessels was significantly higher than that in the DAT gel group (Figure 4B). It is worth noting that the diameter of both groups increased significantly from Week 4 to Week 12.

Immunofluorescence staining of α‐SMA and CD31 revealed that mature blood vessels were different from CD31‐positive blood vessels. From Week 4 to Week 12, the number of α‐SMA positive vessels increased. Similarly, compared with the DAT hydrogel group, t‐DP gel group also showed rich mature vessels (Figure 4C). We believe that the long‐term sustained release of the t‐DP gel growth factor VEGF plays an important role in the early regulation of vascular endothelial cells. VEGF interacts with VEGFR2 expressed on the surface of resting endothelial cells to stimulate endothelial cell differentiation and promote angiogenesis, which explains why the number of CD31‐positive vessels is significantly higher than that of the DAT gel group, and the long‐term sustained release of PDGF regulates the function of pericytes. Peripheral cells are recruited around newly formed blood vessels, and peripheral cells further stabilize new blood vessels and promote vascular maturation by secreting angiopoietin‐1 (Ang‐1), which is also in t‐DP gel. The reason for the increase in mature blood vessels labeled with α‐SMA and CD31. In conclusion, the above results indicate that t‐DP gel promotes adipose tissue regeneration by promoting vascular regeneration and maturation, and the continuous release of growth factors may be an important reason.

## 4 Discussion

Autologous fat grafting has become a widely employed method for reconstructing soft tissues, and one of its important applications is in the reconstruction following tumor resection, making safety an area of significant concern. Recent studies evaluating the long‐term safety of fat grafting have found no increased risk of tumor recurrence, allowing for the confident use of fat grafting in soft tissue reconstruction after tumor surgery [26]. Another area of focus is how to improve the retention rate of fat grafting. In recent years, autologous fat grafting techniques have seen significant advancements. The addition of PRP is believed to enhance the survival and regenerative capacity of fat grafts by releasing growth factors that promote angiogenesis and tissue repair [27]. In terms of operational techniques, minimal manipulation has been shown to minimize damage to adipocytes, thereby increasing the success rate of transplantation [28, 29, 30, 31, 32]. Research on fat scaffolds has demonstrated that optimizing their structure and composition can significantly improve the outcomes of fat grafting [33]. However, some patients lack sufficient autologous fat for transplantation, necessitating the search for alternative materials for soft tissue regeneration.

DAT has attracted attention due to its potential clinically transformation tissue engineering applications, with a wide range of sources, including adipose tissue discarded during abdominal plastic surgery, liposuction, body contouring, and breast reduction surgery. Previous research has focused on different methods of DAT preparation and made significant progress. Different forms of DAT, including hydrogels and powders, are also widely used as adipose tissue regeneration resources and mesenchymal stem cell (MSC) delivery platforms. How to improve the overall performance of DAT has attracted widespread attention from researchers.

Flynn demonstrated that DAT hydrogels supplemented with ADSCs showed increased expression of key regulators of adipogenesis [34]. In vivo studies using subcutaneous DAT grafts in mouse with or without ADSC supplementation have shown that seed DATs exhibit a higher degree of cellular and angiogenic activity compared to nonseed grafts. Previous researchers have shown that adding stem cells, such as ADSCs, is an important strategy for promoting DAT adipogenesis and other functions. However, the application of stem cells has not yet been approved in many countries and fields, greatly limiting their current application. Therefore, researchers conducted further experiments, paying attention to adding cross‐linking agents and active substances to promote angiogenesis and regeneration and regulate the immune response to DAT.

Cross‐linking appears in various forms throughout the literature as a method of enhancing the beneficial properties of the acellular adipose tissue matrix. The cross‐linking of heparin with the DAT matrix was achieved using 1‐ethyl‐3‐[3‐dimethylaminopropyl] carbodiimide (EDC) and N‐hydroxysuccinimide to produce an enhanced DAT matrix as a delivery carrier for basic fibroblast growth factor (bFGF). Compared to using DAT matrix alone in the mouse model, the enhanced DAT matrix resulted in the formation of highly vascularized adipose tissue after 6 weeks. In addition, the implanted DAT matrix exhibited higher levels of CEBP α. The expression of adiponectin and glucose transporter‐4, a key component of adipogenesis. An injectable DAT cross‐linked with hexamethylisocyanate and EDC has been shown to improve the volume retention rate of DAT due to its ability to support the growth and differentiation of ADSCs in vitro.

In addition, in vivo graft models indicate that cross‐linking increases the resistance of DAT to enzyme degradation, which promotes the migration of host cells with adipose tissue development and vascularization without the need to supplement ADSCs. Long‐term research has shown that injectable cross‐linking develops into recently formed adipose tissue with good angiogenesis. Compared with DAT alone, DAT gel supplemented with ADSCs or the cross‐linking agent transglutaminase (TG) showed an improved effect on soft tissue filling. Interestingly, after adding TG to DAT gel, the effect of neovascularization and soft tissue filling is as effective as ADSCs. The above research results indicate that adding cross‐linking agents to DAT is a good strategy for promoting its vascularization and adipogenesis and has broad application prospects.

Here, we have developed an injectable t‐DP gel consisting of DAT and t‐PRP based on collagen‐fibrin interpenetration. In t‐DP gel, PRP is widely used and effective in clinical practice. It is not only a cross‐linking agent but also an active ingredient to promote angiogenesis. t‐DP gel flows at a low temperature of 4°C, and can be injected smoothly through a 27G needle. At a temperature of 37°C, it is cross‐linked and turned into a solid, making it very suitable for soft tissue filling. The stability of DAT, t‐PRP, and t‐DP gels was compared. With the passage of time, t‐DP gel was the most stable. To confirm whether the enhancement of stability is at least partially attributed to the mutual penetration of collagen‐fibrin, we conducted an electron microscopy examination and found that this is indeed the case. In addition, the adipose tissue regeneration of DAT hydrogel is a long process in vivo, in which the function of promoting angiogenesis plays an important role. Consistent with our previous findings, t‐DP gel could achieve sustained release of a variety of growth factors, suggesting that it could continue to promote angiogenesis, which is conducive to adipose tissue regeneration.

In vitro experiments show that our new hydrogel, t‐DP gel, has good potential to promote adipose tissue regeneration. In order to verify the effect of t‐DP gel on adipose tissue regeneration in vivo, we conducted a graft experiment using a mouse model. The graft volume, weight, and adipose tissue regeneration rate of t‐DP gel were greatly improved, and DAT alone showed a poor adipose tissue regeneration rate, as previously reported. Angiogenesis assessed by CD31 immunofluorescence staining and in t‐DP gel compared with DAT alone The mature blood vessels evaluated by α‐SMA immunofluorescence staining showed a significant increase.

Moreover, except for DAT, other tissue‐specific acellular matrices have broad application prospects in tissue regeneration, including the bladder, muscles, skin, small intestinal submucosa, meniscus, and nerves, as they form the cytoskeleton of tissues and organs, support and connect tissue structures, regulate tissue development, and provide the microenvironment required for cell growth. It is worth noting that, like adipocyte extracellular matrix, the main components of tissue extracellular matrix are collagen, fibrin, proteoglycans, and various growth factors. Enhancing the stability of gel and promoting angiogenesis play an important role in tissue regeneration. Therefore, the preparation method of t‐DP gel based on collagen‐fibrin interpenetration can also be used for regeneration of other tissues composed of extracellular matrix hydrogel and t‐PRP.

Of course, our study also has certain limitations. The mouse model used in our research may differ from the outcomes and regeneration processes in humans. Moreover, since the ultimate goal of experimental work is to serve clinical translation, it is crucial to conduct clinical and preclinical studies. To achieve this, we plan to initiate a series of prospective clinical trials to evaluate the effects and safety of t‐DP gel in promoting adipose tissue regeneration in human subjects. These studies should include extensive testing across different patient populations, as well as long‐term follow‐up to monitor the durability of the grafting effects and any potential adverse reactions. Randomized controlled trials should be considered to compare the effectiveness of t‐DP gel with traditional fat grafting techniques, thereby providing higher level evidence for clinical practice.

Additionally, there are challenges in translating hydrogel technologies into clinical applications. One of the primary challenges is achieving manufacturing scalability. It is crucial to ensure that the production process can be scaled up to meet clinical demands while maintaining consistency and quality [35]. This involves addressing several issues, such as the availability of raw materials, production efficiency, and compliance with regulatory standards. Cost‐effectiveness is a significant consideration. The development and production of hydrogels must be economically viable to facilitate widespread adoption in clinical settings. This encompasses not only the direct costs associated with materials and manufacturing but also the indirect costs related to regulatory approval and clinical trials [36]. Furthermore, the biological variability between in vitro and in vivo environments presents challenges in ensuring the safety and efficacy of hydrogels in humans. Extensive clinical trials are necessary to validate their performance and safety profile, which can be time‐consuming and resource‐intensive [37]. Addressing these challenges is essential for the successful translation of t‐DP gel technologies into clinical practice.

## 5 Conclusion

Compared with traditional DAT, the t‐DP gel promotes adipose tissue regeneration through reinforced stability and sustained release of growth factors that promote angiogenesis. The t‐DP gel has great potential applications in the field of plastic surgery for its simple preparation, injectable bioactivity, and regeneration ability. Moreover, the preparation method of t‐DP gel, based on collagen‐fibrin interpenetrating, could also be used for regeneration of other tissues with the composition of extracellular matrix hydrogels and t‐PRP. In summary, our research is of great significance and inspiration for tissue regeneration.

## Author Contributions

Mengmeng Hou, Jiezhang Tang and Yajie Guo contributed to the drafting and editing of the manuscript. Han Peng, Baoyan Liang, Yi Cheng, Zhaoxiang Zhang and Siming Wei contributed to conception and design, or acquisition of data, or analysis and interpretation of data. Huichen Li and Chenggang Yi contributed to revising it critically for important intellectual contentand and given final approval of the version to be published.

## Disclosure

The authors have nothing to report.

## Ethics Statement

All animal experiments were approved by the Ethics Committee of the Fourth Military Medical University (No. IACUC‐20240726). Human tissue collection involved in this research were approved by the Ethics Committee of the Fourth Military Medical University (No. KY20243548‐1).

## Conflicts of Interest

The authors declare no conflicts of interest.

## Data Availability

The data that support the findings of this study are available from the corresponding author upon reasonable request.
